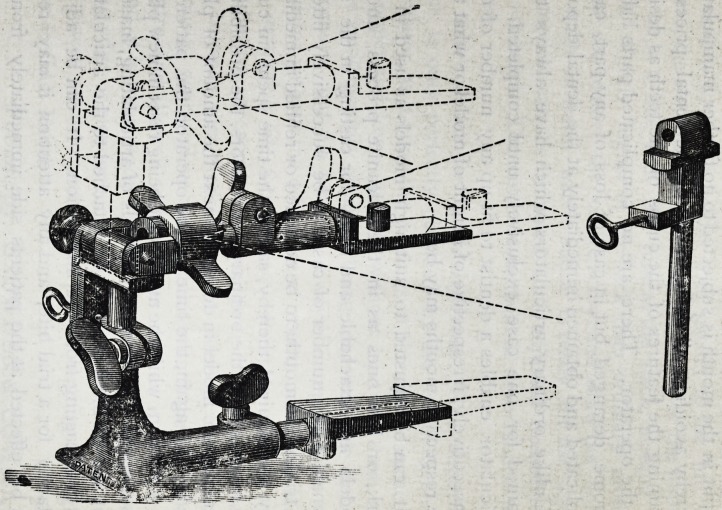# Dr. Genese's Articulator

**Published:** 1883-12

**Authors:** 


ARTICLE II.

### DR. GENESE'S PATENT ARTICULATOR, WITH SET OR LOCK PIN AND INTERCHANGEABLE MODEL HOLDERS.

In calling the attention of the dental profession to my improved articulator, I wish particularly to point out the great advantages of the *lock pin arrangement*, whereby the models can be set permanently at any angle or position; so that an alteration of the like cannot possibly occur after the operator or assistant has once set it, and the teeth can be locked in position while a registration is made for future reference.

Where several articulators are necessary in one workshop this has the advantage over all others as either assistant can affix his models to any articulator, although they may not have been cast on the one in use.

The models can be cast on to the articulator direct from the impression taken from the mouth and one operation allows a trial bite to be ready, immediately the wax composition, or plaster is removed from the model, and a permanent articulator can be obtained at the first visit of the patient.

The operator can set the bite immediately, and without the use of plaster, it can then be *locked* in position and kept so while the case is being set up, or instantly unlocked during the process if desired, and a permanent register kept for future use, should the instrument be wanted for another case.

The position can be changed in fourteen different ways, besides affording every possible range in all those several motions.

By its use the work is so accurately done, that when ready to put in the mouth is subjected to no further manipulations, thereby avoiding all defacements of the dental prices, and retaining the features of the cusps of the teeth as designed by the operator. There are no complicated parts liable to become deranged, but in case of loss of any part, can be duplicated and obtained immediately at the dental depot.

Unlike ordinary articulators which have always to be retained for one case exclusively until completed. This articulator enables a dentist to fit up any number of cases, consecutively, irrespective of height of model or what angle the respective mouths may be.

It can be adjusted to suit high models as used in plate work, or low ones as in the vulcanite process, and the models are detachable and ready for casting in the sand. It enables any number of cases to be successively fitted up for trial in the mouth, to be detached or refilled immediately, they are required, thereby, saving the time spent in cutting the models from the ordinary articulators, the possibility of breaking the model in doing so, next the amount of plaster used ; and again, the time of the operator, the patient and the workman, while the models are being reset in new plaster. It supercedes all existing methods of articulating, inasmuch as it allows the models to be attached to the articulator at the first casting from the impression. It can be adjusted instantly for a trial bite, and any attention it may require can be effected at the patients side immediately from the mouth, locked in that position until the teeth are mounted, and during the alteration of the bite, the models used need not to be removed from the articulator. This is one of the leading features of the instrument. It is a safeguard for the workman, as no amount of fairly rough usage can alter the position of the bite when once screwed up, it is a valuable check on all mistakes in fitting up a case.

DIRECTIONS FOR USING DR. GENESE'S LOCK PIN ARTICULATOR.

Detach the model holder, leaving the centre screw in, paint with non-adhesive, and arrange on a board with the tube pointing away from the workman, after filling the impression, cover the holder with plaster to the hilt and reverse, the tray uppermost. Let the centre of the impression be in a line with the tube at the back, making the model slightly higher in the back than ordinary models. When set, remove the centre screw and draw the holder out, wash in warm water, and it is ready for use again. The models can be trimmed and adjusted immediately they are hard. The entire instrument is never soiled with plaster.

*To secure a bite for future reference.* This important matter is only perfectly obtained by the use of Genese's Articulator, as it takes the bearings of the entire surface of both upper and lower models, without injury to them, and only a small quantity of plaster being used very little shrinkage occurs, and the bite can always be replaced on the articulator without the difference of 1000th part of an inch.

*To arrange a bite for reference.* Paint the models with non-adhesive, mix some plaster and pour in tissue paper, and place between the models that are perfectly articulated previously, gently close the articulator until the pin enters its centre and allow it to harden, as soon as it is set, trim up and it is then ready for any future work. Any overlapping edge or slender tooth may have a little wax or soft paper placed on it to prevent the plaster binding too tight.

Price in Gum Metal, - - - $10

For Sale by SNOWDEN & COWMAN,
BALTIMORE.

## Figures and Tables

**Figure f1:**